# Laparoscopic Nephroureterectomy in a Patient With Hypofunctional Left Kidney and History of Ipsilateral Partial Nephrectomy: A Case Report and Review of the Literature

**DOI:** 10.7759/cureus.76964

**Published:** 2025-01-05

**Authors:** Theodoros Spinos, Vasileios Tatanis, Angelis Peteinaris, Evangelos Liatsikos, Panagiotis Kallidonis

**Affiliations:** 1 Department of Urology, University Hospital of Patras, Patras, GRC; 2 Department of Urology, Medical University of Vienna, Vienna, AUT

**Keywords:** endourology, minimally-invasive surgery, nephroureterectomy, ureteroscopy, utuc

## Abstract

Upper tract urothelial cancer (UTUC) represents a rare malignant urothelial disease arising from the inner surface of the ureter, the calyces or the renal pelvis. This case report describes a 67-year-old patient with a hypofunctional left kidney who was diagnosed with UTUC. This patient had undergone ipsilateral open partial nephrectomy for renal cell carcinoma two years ago. The peculiarities of our case are the therapeutic dilemma of selecting kidney-sparing surgery or radical nephroureterectomy (RNU) for high-risk UTUC in a single functioning kidney and the implementation of a laparoscopic RNU in an already operated field, rendering the operation extremely challenging. Diagnostic ureteroscopy (URS) revealed a large tumor in the right renal pelvis which extended and filled the upper calyces. Three weeks after the diagnostic URS, the patient underwent laparoscopic RNU. The total operative time was 2 hours and 36 minutes, while the estimated blood loss was 150 ml. Postoperatively the creatinine count ranged from 2.6 to 5.1 mg/dL. In this case report, laparoscopic RNU was feasible and effective for a patient with UTUC who has undergone previous kidney intervention. Although the endoscopic management of even high-risk UTUCs must be considered in patients with hypofunctional or single-functioning kidneys, the oncological safety of the patient must be also taken into consideration. The discussion with the patient about the advantages and the drawbacks of both approaches, and his perspective about quality of life are key points for the selection of the therapeutic approach.

## Introduction

Upper tract urothelial cancer (UTUC) is a rare malignancy, comprising only 5-10% of urothelial carcinomas. It originates from the urothelial lining of the renal pelvis, calyces, or ureter and shares a similar pathogenesis with bladder urothelial carcinoma [1]. Diagnosis is usually made with computed tomography (CT) or magnetic resonance (MR) urography. Diagnostic ureteroscopy (URS) with a flexible ureteroscope can confirm the diagnosis, by directly visualizing the lesion. The tumor’s size, morphology and exact location should be carefully defined. Open radical nephroureterectomy (RNU) with bladder cuff excision is the gold standard treatment for high-risk UTUC, while laparoscopic RNU is also a safe approach when performed in experienced centers. Kidney-sparing surgery, such as endoscopic ablation, has gained popularity over the last few years for the treatment of low-risk UTUC [2]. In this case report, we present the case of a 67-year-old patient with a hypofunctional left kidney who was diagnosed with UTUC. This patient had undergone ipsilateral open partial nephrectomy for renal cell carcinoma (RCC) two years ago and was treated with laparoscopic RNU in our center. The peculiarities of our case are the therapeutic dilemma of selecting kidney-sparing surgery or RNU for high-risk UTUC in a patient with a hypofunctional left kidney and the implementation of a laparoscopic RNU in an already operated field, rendering the operation extremely challenging.

## Case presentation

**Figure 1:**
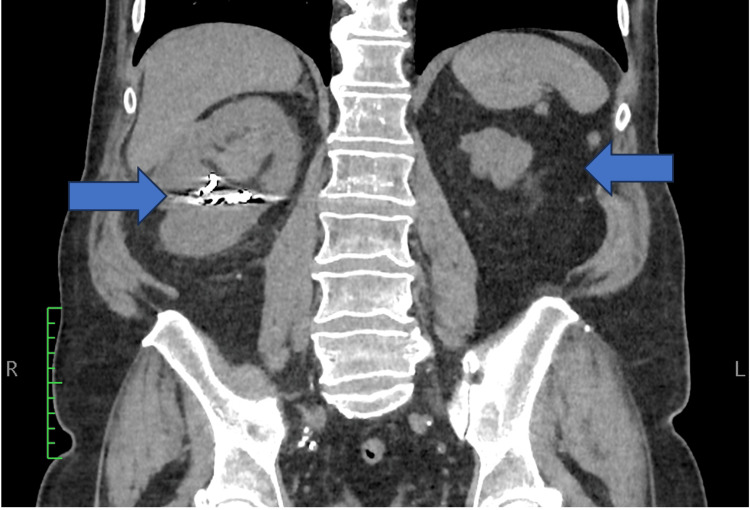
The preoperative CT scan of the patient showing left renal dysgenesis and a right kidney which has undergone previous partial nephrectomy (with clips on it).

A 67-year-old patient was referred to our center for macroscopic haematuria. The patient had a hypofunctional left kidney. A nuclear renal dimercaptosuccinic acid (DMSA) scan documented 25% function of the left kidney and 75% function of the right kidney. The patient had undergone a right-sided open partial nephrectomy for the treatment of RCC two years ago. This RCC was a Fuhrman Grade 2 clear cell carcinoma which was staged as pT3a according to TNM Classification. At the time of the presentation at our ambulatory service, the hemoglobin count of the patient was 7.6 mg/dl, he was admitted to the Urology Department and was transfused with one unit of packed red blood cells (pRBCs). A three-way 22 Fr Foley catheter was placed and continuous bladder irrigation (CBI) was initiated. A kidney-ureters-bladder (KUB) ultrasound (US) documented dilatation of the right pelvicalyceal system, while a non-contrast CT excluded the diagnosis of urolithiasis (Figure 1). CT urography was not performed because the renal function of the patient was impaired (Cr: 2.6 mg/dL, normal range of our laboratory: 0.9-1.6 mg/dl). A cystoscopy revealed blood ooze from the right ureteral orifice. The mucosa of the bladder was normal, excluding the diagnosis of bladder cancer. A selective urine sample from the right ureter was sent for cytologic analysis, revealing high-grade urothelial carcinoma (HGUC), which was categorized as 5 according to the Paris System (TPS V). As suggested by the EAU guidelines, we decided to proceed with diagnostic URS [2].

Under general anesthesia, the patient was placed in a lithotomy position and a hydrophilic guidewire was placed on the right upper urinary tract. A second safety hydrophilic-stiff guidewire was placed using a dual-lumen ureteral catheter and a semi-rigid ureteroscopy was performed, followed by the insertion of a 9.5/11.5Fr ureteral access sheath which was positioned over the working stiff guidewire. After passing a flexible ureterorenoscope through the access sheath, a pyeloscopy was carried out. The ureterorenoscope used was a single-use flexible PU3033A (PUSEN Medical, Shenzhen, China). Diagnostic URS revealed a large tumor in the right renal pelvis which extended and filled the upper calyces. A sample biopsy was taken, indicating urothelial carcinoma. This tumor was considered as high-risk UTUC, according to the EAU guidelines, being obviously larger than 2 cm, as it was also documented by urine cytology and the sample biopsy [2]. Due to the large size of the tumor, we considered RNU as the safest choice for the patient, from an oncological point of view. The patient was informed about the benefits and the drawbacks of both approaches and he decided to undergo RNU, being informed about the possibility of becoming dialysis-dependent, prioritizing his oncological safety.

**Figure 2:**
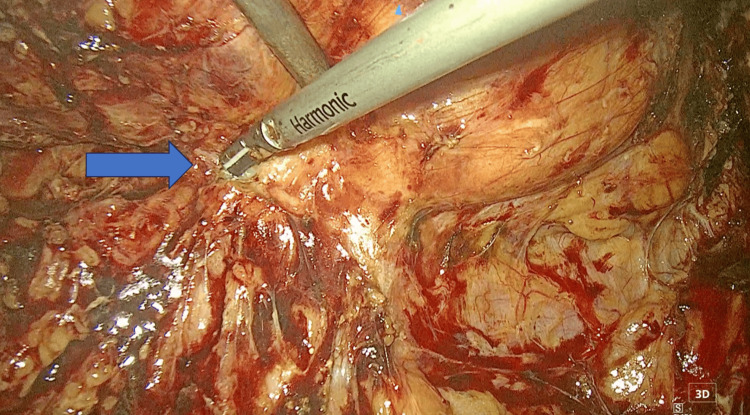
Endoscopic view of the right kidney during laparoscopic nephroureterectomy, showing extensive adhesions and fibrosis around it.

Three weeks after the diagnostic URS, the patient underwent laparoscopic RNU. Before RNU the patient underwent staging with a CT scan of the chest, MRI scan of the upper and lower abdomen and bones scan. Under general anesthesia, the patient was placed in the lateral decubitus position. A 10 mm Hasson trocar was placed at the lateral margin of the rectus abdominis muscle at the level of the umbilicus for the insertion of the camera. A 12 mm trocar and a 5 mm trocar were placed at the level of the mid-clavicular line, forming a trigone with the camera trocar. After entering into the peritoneal cavity, extensive adhesiolysis was performed, followed by identification of the ureter and the hilar structures (Figure 2). The ureter was proximally ligated with a clip and the renal vessels were ligated and dissected using a stapler, followed by dissection of renal attachments outside the Gerota’s fascia. After placement of an additional 12 mm trocar, the ureter was dissected down to the level of the ureterovesical junction and the bladder cuff was excised (totally intracorporeally). The total operative time was 2 hours and 36 minutes, while the estimated blood loss was 150 ml. Postoperatively the creatinine count ranged between 2.6 and 5.1 mg/dL. The patient was disease-free during the last follow-up, as evidenced by a negative cystoscopy, negative urine cytology, negative CT urography and negative chest CT. Moreover, although the patient was dialysis dependent (twice per week), he was satisfied with his quality of life.

## Discussion

This case is a chance to discuss three interesting topics: 1) the selection of RNU or kidney-sparing approaches for UTUC management in special conditions, such as in hypofunctional or single-functioning kidneys, 2) the feasibility, the safety and the efficacy of laparoscopic RNU in the retreatment setting after previous interventions and 3) the existence of RCC and UTUC in the same patient. Although RNU is the standard treatment for high-risk UTUCs (such as in our case, tumor > 2 cm), the EAU guidelines state that kidney-sparing management should also be considered in high-risk patients with imperative indication on a case-by-case basis [2]. Kawada et al., in a recently performed systematic review and meta-analysis comparing the oncological and safety outcomes between endoscopic management and RNU, reported that the endoscopic management showed comparable survival outcomes with the RNU at the cost of higher recurrence rates, though [3]. The authors concluded that although the endoscopic management is an excellent choice for selected patients, these patients should be well informed about the need of following a strict surveillance program and the risk of additional interventions [3]. Alam et al., in a very interesting study, compared the survival outcomes between the endoscopic management of UTUC versus RNU in 2108 solitary kidney patients with localized disease [4]. The authors concluded that endoscopic management and RNU were associated with comparable survival outcomes, highlighting that the potential benefits of RNU in these patients may not outweigh the risks of becoming anephric and therefore dependent on dialysis [4]. However, in both studies, only low-risk UTUCs were included [3,4]. In our case, the tumor was large (around 4 cm), while urine cytology documented high-grade UTUC, and thus we decided to perform RNU for the oncological safety of the patient at the cost of permanent dialysis dependence. The implementation of well-designed studies, comparing kidney-sparing approaches and RNU for high-risk UTUCs, is of utmost importance.

Chen et al. compared RNU and endoscopic management for localized UTUC in a high endemic region with high-grade-predominant disease. They concluded that both approaches showed similar overall survival, cancer-specific survival and intravesical recurrence-free survival, but the endoscopic approach was associated with worse disease-free survival. Interestingly, the authors supported that although the endoscopic approach may preserve more renal units and delay the appearance of kidney failure, it does not exclude the possibility of dialysis dependence in the long term [5]. Finally, Giulioni et al. recently performed a systematic review and meta-analysis (World Journal of Urology, 2024) of comparative studies, regarding RNU versus endoscopic intervention in localized UTUC. They concluded that although the endoscopic management and RNU show almost comparable oncological outcomes for patients with localized UTUC, RNU was associated with greater 5-year overall survival in patients with high-grade UTUC [6].

Regarding the feasibility and safety of laparoscopic RNU in the retreatment setting, to the best of our knowledge, there are no reports in existing literature. However, there is limited evidence regarding the feasibility of laparoscopic nephrectomy after previous partial nephrectomy. Shah et al. investigated the feasibility and the outcomes of laparoscopic nephrectomy in patients who have undergone previous partial nephrectomy for RCC and who presented with locoregional disease recurrence [7]. They concluded that a laparoscopic approach in these cases is technically demanding and associated with significant perioperative morbidity and a high risk of open conversion. According to the authors, careful patient selection is the key to optimize the outcomes. In our case, extensive adhesiolysis was necessary while recognition of the kidney was challenging. However, the case was completed in a totally intracorporeal approach [7].

Finally, regarding the existence of RCC and UTUC in the same patient there is limited data in the existing literature. Qi et al. presented a large series of 27 cases with concurrent RCC and urothelial carcinoma and reported the long-term outcomes. Interestingly, the authors reported that the coexistence of these tumors is very rare, that treatment should be individualized and that the prognosis of the patients is potentially determined by the most aggressive tumor [8].

## Conclusions

In this case report, laparoscopic RNU was feasible and effective for a patient with UTUC who has undergone previous kidney intervention. Although the endoscopic management of even high-risk UTUCs must be considered in patients with hypofunctional or single-functioning kidneys, the oncological safety of the patient must be also taken into consideration. The discussion with the patient about the advantages and the drawbacks of both approaches, and his perspective about quality of life are key points for the selection of the therapeutic approach. The implementation of additional well-designed studies comparing kidney-sparing techniques and RNU in hypofunctional or single kidney patients is of utmost importance, so that safe conclusions can be drawn.
